# β-Arrestin 1 in Thyrotropin Receptor Signaling in Bone: Studies in Osteoblast-Like Cells

**DOI:** 10.3389/fendo.2020.00312

**Published:** 2020-05-20

**Authors:** Alisa Boutin, Marvin C. Gershengorn, Susanne Neumann

**Affiliations:** Laboratory of Endocrinology and Receptor Biology, National Institute of Diabetes and Digestive and Kidney Diseases, National Institutes of Health, Bethesda, MD, United States

**Keywords:** β-arrestin 1, TSH receptor, IGF1 receptor, osteoblast-like cells, positive allosteric modulator, crosstalk

## Abstract

A direct action of thyrotropin (TSH, thyroid-stimulating hormone) on bone precursors in humans is controversial. Studies in rodent models have provided conflicting findings. We used cells derived from a moderately differentiated osteosarcoma stably overexpressing human TSH receptors (TSHRs) as a model of osteoblast precursors (U2OS-TSHR cells) to investigate TSHR-mediated effects in bone differentiation in human cells. We review our findings that (1) TSHR couples to several different G proteins to induce upregulation of genes associated with osteoblast activity—interleukin 11 (IL-11), osteopontin (OPN), and alkaline phosphatase (ALPL) and that the kinetics of the induction and the G protein-mediated signaling pathways involved were different for these genes; (2) TSH can stimulate β-arrestin-mediated signal transduction and that β-arrestin 1 in part mediates TSH-induced pre-osteoblast differentiation; and (3) TSHR/insulin-like growth factor 1 (IGF1) receptor (IGF1R) synergistically increased OPN secretion by TSH and IGF1 and that this crosstalk was mediated by physical association of these receptors in a signaling complex that uses β-arrestin 1 as a scaffold. These findings were complemented using a novel β-arrestin 1-biased agonist of TSHR. We conclude that TSHR can signal via several transduction pathways leading to differentiation of this model system of human pre-osteoblast cells and, therefore, that TSH can directly regulate these bone cells.

## Introduction

Thyroid-stimulating hormone (TSH), also known as thyrotropin, is a hormone that activates TSH receptors (TSHRs) to stimulate development of the thyroid *in utero* and on thyroid cells to stimulate production of thyroid hormones thyroxine (T4) and triiodothyronine (T3) in the adult. Thyroid hormones are essential for skeletal development and healthy bone metabolism (1, 2). Clinical studies have demonstrated that euthyroid status in the adult is important for bone homeostasis. Hyperthyroidism leads to increased bone resorption which causes reduced bone and its mineralization. Graves' hyperthyroidism presents an increased risk for osteoporosis (3, 4). Patients with hypothyroidism exhibit reduced bone turnover as osteoclast activity is reduced. The effects of hyperthyroidism, hypothyroidism, and subclinical hyperthyroidism on bone metabolism have been extensively reviewed (2, 5, 6).

While the pivotal role of thyroid hormones in bone homeostasis has been well documented, the role of TSH itself is still under study. The question if there is a direct, thyroid hormone-independent action of TSH on bone is of specific interest. TSHR expression has been demonstrated in rodent osteoblasts and osteoclasts (2). TSHR knockout causes osteoporosis and focal osteosclerosis in mice (7). TSH administration inhibits bone loss in adult, ovariectomized rodents consistent with the idea that TSH is an activator of bone formation (8). Overall, *in vivo* studies in mice and rats propose TSH as a fine-tuning regulator of bone homeostasis, and these findings have been comprehensively reviewed (1, 2, 6, 7, 9, 10).

Several clinical studies have also pointed to direct action of TSH on bone. Administration of recombinant human TSH (rhTSH) in postmenopausal women increased serum N-terminal propeptide of type-I procollagen (PINP), a marker of bone formation, demonstrating an anabolic effect of TSH in humans (11). Mazziotti et al. showed that short-term rhTSH stimulation leads to a reversible inhibition of bone resorption in postmenopausal women suggesting a role for TSH in patients with bone loss and a high bone turnover rate (12). Furthermore, epidemiological studies demonstrated a tight relationship between low TSH levels and parameters of bone loss and fracture risk [reviewed in (4)]. However, the role of TSH on the adult skeleton and its mechanisms of action in human bone have yet to be defined in more detail and are still controversial [reviewed in (1, 2, 4, 6)].

TSHR is expressed in human bone, however, TSH is not expressed in primary human osteoblasts or osteoclasts (13). Pituitary TSH works systemically, and therefore, it is likely that it can activate TSHRs in bone. Thyrostimulin, an ancestral glycoprotein hormone and TSHR agonist, has been considered as a regulator of bone formation. In contrast to TSH, thyrostimulin is expressed in osteoblasts and osteoclasts (14). The combination of *in vivo* and *in vitro* studies demonstrated a role for thyrostimulin during skeletal development but an unessential role in the adult skeleton (14).

In human thyrocytes, TSHR coupling to Gα_s_ and activation of the cAMP-protein kinase A (PKA) signal transduction system has been considered the primary pathway of TSH regulation (15). *In vitro* studies in bone cells were initially hampered by the assumption that TSH-induced cAMP production is the primary TSHR-mediated signaling pathway. TSH stimulation of some bone cells did not result in cAMP production, and therefore, at first, a potential physiological role of TSH in bone was underestimated. However, recent studies have shown that other G protein- and β-arrestin-mediated signaling pathways can be activated via TSHR and the quest for the role of TSH in bone metabolism gained traction again. TSHR activates mitogen-activated protein kinase 1/3 (ERK1/2) (16), p38 mitogen-activated protein kinase 1 (p38 MAPK) (17), and AKT serine/threonine kinase 1 (AKT1) (18). The activation of these three kinases by other G protein-coupled receptors (GPCRs) is known to require, in part, β-arrestin involvement (19). Parathyroid hormone (PTH), a key regulator of bone metabolism acting through the parathyroid hormone 1 receptor (PTH1R), translocates both β-arrestin 1 and 2 and activates ERK1/2 (20). Our group has demonstrated that TSH mediates activatory TSHR signaling through β-arrestin 1, and that this pathway plays an important role in stimulating upregulation of osteoblast markers driving the precursors toward an osteoblast phenotype (21).

This review will focus on TSH signaling in human osteoblast precursor cells and summarize the roles of G protein- and β-arrestin-mediated signaling pathways with a special emphasis on the role of β-arrestin 1 in bone physiology.

## G Protein-Mediated TSHR Signaling in U2OS-TSHR Cells

The majority of studies on the TSH effect on bone homeostasis were performed in mice or mouse cell cultures (7, 8, 22–24). We used U2OS cells derived from a moderately differentiated osteosarcoma stably overexpressing human TSHR (U2OS-TSHR cells) as a model of osteoblast precursors (21) to investigate TSHR-mediated effects in bone differentiation in human cells. U2OS cells had previously been shown to exhibit differentiation toward osteoblasts (25). Parental U2OS cells express very low endogenous TSHR; TSHR mRNA in U2OS cells is four orders of magnitude lower than in U2OS-TSHR cells. Treatment of parental U2OS cells with TSH did not stimulate cAMP production or osteoblast marker upregulation (21).

TSHR is known to couple to multiple G proteins (26). This capability to couple to and activate multiple G protein-mediated signaling pathways is also exhibited by the PTH1R (27), which is highly expressed on osteoblasts. PTH1R can signal via several signaling pathways including cAMP to activate PKA and Gα_q/11_ to activate phospholipase C.

We demonstrated that TSHR couples to several different G proteins to induce upregulation of genes associated with osteoblast activity in U2OS-TSHR cells: interleukin 11 (IL-11) (28, 29), osteopontin (OPN) (30), and alkaline phosphatase (ALPL) (31). The kinetics of the induction and the signaling pathways involved, however, were different for these genes (32).

IL-11 upregulation was rapid and reached its maximum within 4 h of treatment with TSH. IL-11 expression was primarily regulated by the Gα_s_-cAMP pathway. The conclusion that TSH regulated IL-11 expression via the Gα_s_-cAMP pathway was based on the observation that forskolin (FSK), an adenylyl cyclase activator, upregulated IL-11, and H-89, a PKA inhibitor, inhibited TSH-mediated IL-11 upregulation. Silencing of Gα_s_ by siRNA inhibited TSH-mediated upregulation of IL-11 as well as basal IL-11 expression, most likely due to inhibition of TSHR's constitutive Gα_s_-mediated signaling activity.

By contrast, we found that OPN induction by TSH was independent of the Gα_s_ pathway as neither FSK nor H-89 had a significant effect. OPN induction was slower than that of IL-11 requiring 72 h for detection. Pertussis toxin (PTX), which inhibits Gα_o/i_ protein activation, inhibited OPN stimulation by TSH. The knockdown of Gα_i1, 2, 3_ confirmed that Gα_i_ is important for TSH-induced OPN upregulation. In addition, we showed that p38 MAPK phosphorylation was necessary for OPN upregulation. This likely occurred downstream of Gα_i_ because TSH-mediated phosphorylation of p38 MAPK was inhibited by PTX, and siRNA knockdown of p38 MAPK and LY2228820, a p38 MAPK kinase inhibitor, caused suppression of OPN also.

We determined that ALPL induction required approximately 24 h for detection and exhibited a biphasic dose response curve with lower doses of TSH resulting in decreased levels of ALPL and higher doses stimulating its upregulation. Interestingly, the EC_50_ for cAMP stimulation was similar to that of downregulation of ALPL. Furthermore, activation of the cAMP pathway by FSK downregulated basal levels of ALPL whereas inhibition of this signaling cascade by H-89 and Gα_s_ knockdown increased basal ALPL levels. Conversely, the EC_50_ for Gα_q/11_ pathway activation was similar to that of ALPL upregulation. We had shown previously that higher doses of TSH were required for Gα_q/11_ activation as two TSH molecules were necessary to bind to the TSHR homodimer in order to activate Gα_q/11_ signaling (33). The knockdown of Gα_q/11_ confirmed that Gα_q/11_ signaling is involved in ALPL upregulation by TSH. We showed that ALPL upregulation was mediated in part by protein kinase C (PKC), which is activated downstream of Gα_q/11_ by diacylglycerol, because the PKC inhibitor GF109203X inhibited ALPL upregulation, and the PKC activator phorbol 12,13-dibutyrate upregulated ALPL. Lastly, since the dose responses for ERK1/2 phosphorylation and ALPL upregulation were similar, we used U0126, a mitogen-activated protein kinase kinase 7 (MEK) inhibitor, to show that ERK1/2 inactivation also inhibited ALPL induction. Combined, our findings indicate that regulation of ALPL by TSH is biphasic. The higher potency (lower doses) TSH effect to downregulate ALPL is mediated by the Gα_s_-cAMP pathway whereas the lower potency (higher doses) TSH effect to upregulate ALPL is mediated in part by the Gα_q/11_-PKC-ERK1/2 signaling cascade.

Thus, only upregulation of IL-11, a cytokine required for normal bone remodeling, was mainly mediated via cAMP, the traditionally recognized “primary” signaling pathway for TSH. By contrast, ALPL was inhibited by cAMP at low TSH concentrations. It appears that unlike in thyrocytes, in which cAMP is a positive modulator of thyroid cell differentiation (34, 35), in U2OS-TSHR cells, cAMP is a negative modulator of differentiation and is only one of several pathways involved in osteoblast differentiation. These findings help us to better understand the multi-pathway nature of TSHR signaling revealing that it is more complex and cell context-dependent than previously understood (32).

Of note, thyrostimulin induced responses similar to those of TSH in regulation of IL11, OPN and ALPL (32). Although it appears that TSH is acting as a hormone to reach the site(s) of action in the bone via the bloodstream, thyrostimulin may play a role in anabolic regulation in bone in a paracrine manner during early skeletal development, as has been demonstrated in mice (14).

## β-Arrestin-Mediated Signaling in U2OS-TSHR Cells

It has been shown that the anabolic effects of PTH are mediated via a β-arrestin signal transduction pathway independent of G protein signaling. In the case of PTH1R, the primary activator of the anabolic effect is β-arrestin 2, which was demonstrated using a β-arrestin-biased agonist (36). In contrast, nonselective PTH also mediates osteoblast-osteoclast coupling via Gα_s_/cAMP promoting bone turnover (37, 38).

**Figure 1:**
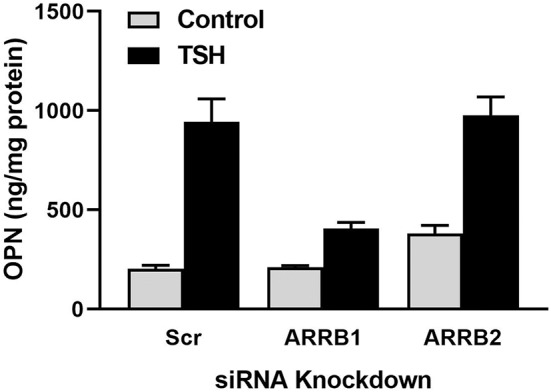
β-arrestin 1, but not β-arrestin 2, mediates OPN secretion. U2OS-TSHR cells were treated with non-targeting (scramble siRNA, Scr), β-arrestin 1 (ARRB1), or β-arrestin 2 (ARRB2) siRNAs for 24 h. After 72 h, the cells were incubated with 2 μM TSH in EMEM supplemented with 0.1% BSA for 5 days prior to the ELISA measurement of OPN secreted into the cell culture media (21).

We investigated whether TSH can stimulate β-arrestin mediated signal transduction in U2OS-TSHR cells (21). In addition to TSH, we utilized a small molecule ligand C2 (NCGC00161870) that is functionally selective for cAMP pathway activation (39). We demonstrated that TSHR stimulation with TSH, but not C2, leads to phosphorylation of ERK1/2, AKT1, and p38 MAPK phosphokinases. Similarly, we found that TSH, but not C2, stimulated upregulation of osteoblast markers such as ALPL, OPN, and tumor necrosis factor superfamily member 11 (RANKL). Using the DiscoveRx PathHunter β-arrestin protein complementation assay, we found that TSH induced translocation of both β-arrestin 1 and 2 to the TSHR while C2, despite being fully efficacious for cAMP production, did not. Even though TSH stimulated translocation of both β-arrestins, siRNA-mediated silencing of β-arrestins demonstrated that only β-arrestin 1 was necessary for phosphorylation of ERK1/2, AKT1, and p38 MAPK phosphokinases. In contrast, we observed an increase in phospho-AKT1 and ERK1/2 with β-arrestin 2 knockdown. Frenzel et al. (40) showed that β-arrestin 2 was predominantly involved in TSHR desensitization, which may explain why β-arrestin 2 knockdown caused an increase in AKT1 and ERK1/2 phosphorylation. Lastly, we examined involvement of β-arrestins in mediation of TSH-induced expression of osteoblastic markers. Knockdown of β-arrestin 1, but not β-arrestin 2, inhibited upregulation of RANKL and OPN. Figure 1 illustrates that the β-arrestin 1 pathway accounts for ~80% of TSH-stimulated OPN upregulation. The remaining 20% is most likely mediated by G proteins, in particular Gα_i_ (32). β-arrestin 2 does not play a role in OPN secretion (Figure 1). Thus, in U2OS-TSHR cells, β-arrestin 1 mediates TSHR-induced activatory signals, whereas β-arrestin 2 appears to be a mediator of inhibitory signals and desensitization. The fact that β-arrestin 1 in part mediates TSH-induced osteoblast differentiation indicates that this pathway may play an anabolic role in bone physiology (21).

The potencies for TSH-induced translocation of both β-arrestins to the TSHR were approximately 16-fold lower than that for cAMP stimulation and similar to that for stimulation of phosphoinositide signaling (33, 41). As mentioned above, we previously demonstrated that these differences in potency could be attributed to the fact that for Gα_s_ coupling leading to adenylyl cyclase activation and cAMP production binding of a single molecule of TSH to the TSHR homodimer (or oligomer) was sufficient. However, coupling to Gα_q/11_ and subsequent activation of phospholipase C and phosphoinositide signaling required binding of two TSH molecules to a TSHR homodimer (33). Even though evidence has been presented that a number of GPCRs form heterodimers that can interact with β-arrestins for internalization, it remains controversial whether homodimerization is required for binding to β-arrestins (42). Nonetheless, to explain the lower potency of TSH to stimulate translocation of β-arrestins to TSHR compared to cAMP stimulation, we suggest that translocation is enhanced by binding of two TSH molecules to a TSHR homodimer (21).

To study TSH-enhanced osteoblast differentiation more deeply, we sought to discover a drug-like agonist that is selective for β-arrestin 1 pathway activation by TSHR. D3-βArr (NCGC00379308) was identified through high throughput screening and selected out of 368,816 compounds because it caused translocation of β-arrestin 1 to the TSHR without stimulation of cAMP production (43). Thus, D3-βArr is a functionally selective TSHR ligand. D3-βArr stimulated β-arrestin 1 translocation more effectively than TSH and potentiated the effect of TSH in stimulating β-arrestin 1 translocation. D3-βArr also stimulated β-arrestin 2 translocation but with decreased potency and efficacy compared to β-arrestin 1. The functional selectivity of D3-βArr allowed us to suggest that this drug-like ligand may be used as a probe to enhance β-arrestin 1-mediated signaling over G protein-dependent signaling in follow-up studies on its role in bone physiology.

**Figure 2:**
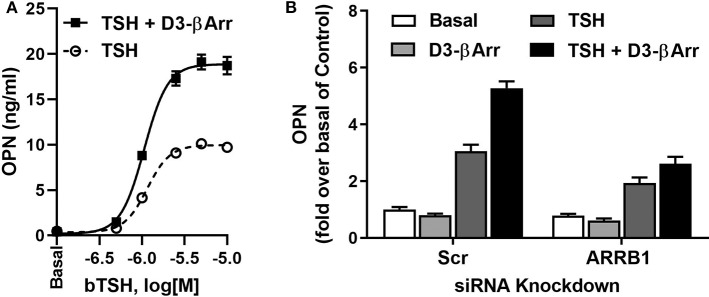
D3-βArr is a functionally selective TSHR agonist. **(A)** D3-βArr is a positive allosteric modulator for TSH-induced OPN secretion. U2OS-TSHR cells were treated with increasing doses of TSH with or without 5 μM D3-βArr in EMEM with 0.1% BSA for 7 days prior to OPN measurement by ELISA in cell culture media (43). **(B)** The potentiating effect of D3-βArr is mediated by β-arrestin 1 in U2OS-TSHR cells. Cells were transfected with non-targeting (Scr) or β-arrestin 1 (ARRB1) siRNA, respectively. After 72 h of siRNA-mediated knockdown, cells were treated with 10 μM D3-βArr, 2 μM TSH or their combination in EMEM with 0.1% BSA for 5 days. OPN secretion was measured in conditioned media by ELISA (43).

Furthermore, we studied whether the effect of D3-βArr on β-arrestin 1 translocation and/or potentiation of TSH would translate into enhancement of TSH-induced upregulation of osteoblast genes. D3-βArr by itself did not increase OPN mRNA but upregulated ALPL mRNA 4-fold. Most importantly, D3-βArr enhanced TSH stimulation of OPN secretion (Figure 2A). siRNA knockdowns of β-arrestin 1 and 2 confirmed that β-arrestin 1 mediated the potentiating effect of D3-βArr on TSH-stimulated OPN secretion (Figure 2B). Thus, we established that D3-βArr is a positive allosteric modulator of TSH-induced upregulation of osteoblast genes and OPN secretion in a human *in vitro* cell model. D3-βArr or an analog may serve to probe TSHR physiology in bone *in vivo* in the future (43).

## TSHR/IGF1R Crosstalk and the Role β-Arrestin 1 as a Scaffold of this Protein Complex in U2OS-TSHR Cells

Crosstalk (or transactivation) between GPCRs and receptor tyrosine kinases is an established signaling mechanism (44, 45). Crosstalk between TSHR and insulin-like growth factor 1 (IGF1) receptor (IGF1R) has recently been studied in the context of the pathogenesis of Graves' ophthalmopathy (46–49) as well as thyroid specific gene upregulation in human primary thyrocytes (50). We examined whether this phenomenon occurred in U2OS-TSHR cells.

**Figure 3:**
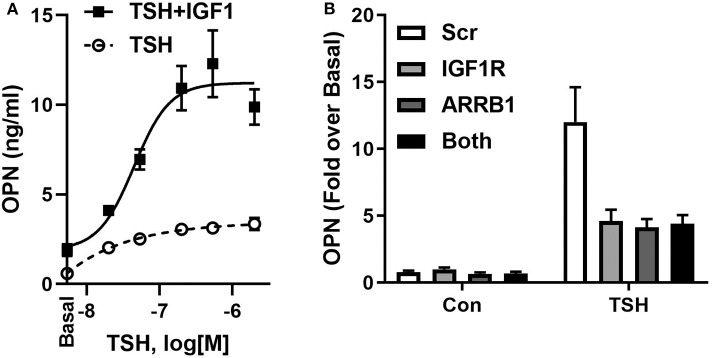
TSHR and IGF1R exhibit crosstalk in U2OS-TSHR cells. **(A)** TSH and IGF1 synergistically stimulate OPN secretion. U2OS-TSHR cells were treated with increasing doses of recombinant human TSH (rhTSH) with or without 100 ng/ml IGF1. After 5 days, OPN secreted into cell culture media was measured by ELISA. This original figure is based on the data published in (52). **(B)** IGF1R and β-arrestin 1 are part of the same signaling pathway in TSHR/IGF1R crosstalk-mediated OPN secretion. U2OS-TSHR cells, in which IGF1R and β-arrestin1 (ARRB1) were silenced separately or simultaneously were treated with 10 mU/ml rhTSH. After 5 days, OPN was measured in culture media and compared to scramble siRNA (Scr) control. Knockdown efficiencies of IGF1R and ARRB1 were 95.5 and 82.9%, respectively (52).

IGF1 and IGF1R were shown to play important roles in bone development and in regulation of bone homeostasis in the adult (51). In U2OS-TSHR cells, we observed that IGF1 alone mildly stimulated OPN but, more importantly, TSH and IGF1 synergized to upregulate OPN secretion (Figure 3A). Individual knockdowns of β-arrestin 1 and IGF1R resulted in inhibition of OPN secretion to similar extents. Their co-knockdown did not show any additive effect (Figure 3B), consistent with the idea that these proteins are in the same signaling pathway. Moreover, the IGF1R inhibitor linsitinib decreased TSH-mediated OPN secretion even in the absence of IGF1 (52). These data support the idea of TSHR/IGF1R crosstalk in U2OS-TSHR cells and suggested a potential role for β-arrestin 1 in this protein complex (52).

**Figure 4:**
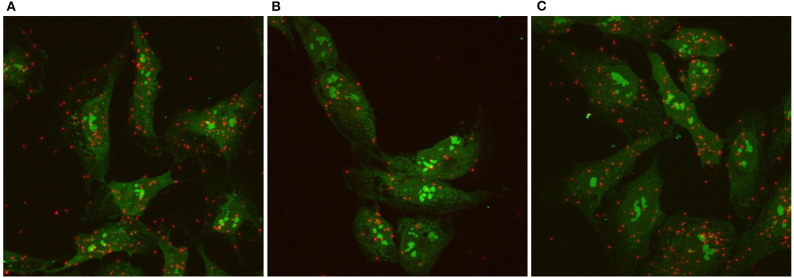
TSHR/IGF1R crosstalk is dependent on receptor proximity and β-arrestin 1 stabilizes the protein complex. TSHR/IGF1R signaling complexes were detected using a Proximity Ligation Assay (PLA) and visualized with fluorescent microscopy. A PLA signal is measured when TSHR and IGF1R are in a protein complex within 40 nm of each other. PLA was performed in unstimulated U2OS-TSHR cells treated with non-targeting **(A)**, β-arrestin 1 (ARRB1) **(B)**, or β-arrestin 2 (ARRB2) **(C)** siRNA. Positive PLA signals are shown in red. SYTO9 nucleic acid staining, applied to determine the cell number, is shown in green at ×63 magnification. The knock-down of β-arrestin 1 reduced the number of positive PLA signals by 55% demonstrating this proteins stabilizing function in the TSHR/IGF1R complex (52).

**Figure 5:**
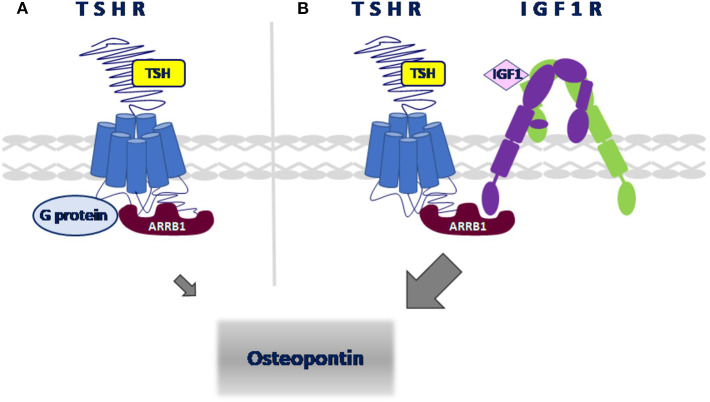
Model of signaling pathways leading to OPN secretion in U2OS-TSHR cells. **(A)** TSHR-mediated upregulation of OPN. TSH-induced OPN secretion is regulated via Gα_i_ and β-arrestin 1-mediated signal transduction pathways. **(B)** β-arrestin 1 scaffolds TSHR and IGF1R to enable crosstalk. TSH and IGF1 synergistically upregulate OPN secretion. β-arrestin 1 stabilizes the receptor complex which enables TSHR/IGF1R crosstalk.

We used the Proximity Ligation Assay (PLA) to show that TSHR and IGF1R were held within 40 nM or less of each other by β-arrestin 1. Knockdown of β-arrestin 1 in U2OS-TSHR cells exhibited a 55% decrease (^*^P < 0.0001) in PLA-positive signals per cell while knockdown of β-arrestin 2 had no effect (Figure 4). These results provide evidence that β-arrestin 1 is associated with TSHR and IGF1R under unstimulated conditions. Moreover, since β-arrestin 1 knockdown inhibited the synergistic stimulation of OPN secretion, these data demonstrate that close proximity of TSHR and IGF1R is required for receptor crosstalk, and that this physical proximity in a protein complex depends on the presence of β-arrestin 1. Figure 5 illustrates a model of TSHR-mediated OPN secretion mediated by G proteins and β-arrestin 1 as well as the role of β-arrestin 1 as a central scaffold in allowing for TSHR/IGF1R crosstalk leading to further increase of OPN secretion and U2OS-TSHR cell differentiation (52).

## Conclusion

In this review, we summarized our findings showing that the role of β-arrestins in TSHR signal transduction in osteoblast-like cells is more prominent than previously understood. In addition to canonical receptor desensitization and internalization, β-arrestin 1, along with G proteins, mediates TSH-enhanced osteoblast differentiation. Specifically, β-arrestin 1 serves as a physical scaffold to enable TSHR/IGF1R crosstalk (Figure 5). We also describe how the β-arrestin 1-biased agonist D3-βArr, a small molecule positive allosteric modulator for TSHR, may be used to better understand the role of TSHR in bone differentiation. Since TSHR may signal via β-arrestin 1 in humans in a clinically relevant manner, our data suggest that a TSHR positive allosteric modulator may have a potential role as a therapy for osteoporosis.

## Author Contributions

All authors have contributed significantly to the work, have read the manuscript, attest to the validity and legitimacy of the data and its interpretation, and agree to its submission.

## Conflict of Interest

The authors declare that the research was conducted in the absence of any commercial or financial relationships that could be construed as a potential conflict of interest.
